# Pyrosequencing Dried Blood Spots Reveals Differences in HIV Drug Resistance between Treatment Naïve and Experienced Patients

**DOI:** 10.1371/journal.pone.0056170

**Published:** 2013-02-07

**Authors:** Hezhao Ji, Yang Li, Binhua Liang, Richard Pilon, Paul MacPherson, Michèle Bergeron, John Kim, Morag Graham, Gary Van Domselaar, Paul Sandstrom, James Brooks

**Affiliations:** 1 National HIV & Retrovirology Laboratories, National Microbiology Laboratory, Public Health Agency of Canada, Ottawa, Canada; 2 Bioinformatics Core, National Microbiology Laboratory, Public Health Agency of Canada, Winnipeg, Canada; 3 Department of Medical Microbiology, University of Manitoba, Winnipeg, Canada; 4 Division of Infectious Diseases, The Ottawa Hospital, Ottawa, Canada; 5 Genomics Core Facility, National Microbiology Laboratory, Public Health Agency of Canada, Winnipeg, Canada; University of Amsterdam, The Netherlands

## Abstract

Dried blood spots (DBS) are an alternative specimen collection format for HIV-1 genotyping. DBS produce HIV genotyping results that are robust and equivalent to plasma when using conventional sequencing methods. However, using tagged, pooled pyrosequencing, we demonstrate that concordance between plasma and DBS is not absolute and varies according to viral load (VL), duration of HIV infection and antiretroviral therapy (ART) status. The plasma/DBS concordance is the highest when VL is ≥5,000 copies/ml and/or the patient has no ART exposure and/or when the duration of HIV infection is ≤2 years. Stepwise regression analysis revealed that VL is most important independent predictor for concordance of DBS with plasma genotypes. This is the first study to use next generation sequencing to identify discordance between DBS and plasma genotypes. Consideration should be given to VL, duration of infection, and ART exposure when interpreting DBS genotypes produced using next generation sequencing. These findings are of particular significance when DBS are to be used for clinical monitoring purposes.

## Introduction

HIV genotyping identifies genotypic markers of drug resistance (DR), assesses HIV diversity, and provides data for molecular epidemiological and evolutionary analysis [1]. Conventional HIV genotyping is performed using Sanger sequencing (SS) with plasma or serum as starting material. With the ease of collection, processing, transportation and simplified storage conditions, dried blood spots (DBS) present an alternative specimen collection format for HIV genotyping especially in resource limited settings [2]–[5].

Plasma HIV genotyping results are derived from the RNA contained within the cell-free circulating virus. In contrast, template material contained in DBS consists of RNA from circulating virus and DNA from cell-associated, integrated provirus. In comparison to circulating viruses, the provirus population represents a dynamic history of the virus; each reflecting the selection pressures and adaptations at the time of integration. For example, the dynamics of early infection [6], host selection pressure or antiretroviral therapy (ART) may eliminate less-fit viral variants from circulation but the footprints of these “failing” strains may become embedded in the proviral genotype. Using Sanger sequencing the two viral populations can be demonstrated to be fairly homogenous, however, sequence divergence has been shown to modestly increase over time. [6], [7] [8] . Numerous manuscripts have attributed equivalency to DBS genotypes obtained from both drug naïve and ART experienced patients [2], [9]–[11]. However, papers have also identified divergence between circulating and proviral population genotypes in both ART experienced and ART naïve patients [12]–[15]. Given the potential divergence of amplifiable templates contained in DBS, it is possible that if examined with sufficient resolution, the plasma and DBS genotypes may differ. Tagged, pooled-pyrosequencing (TPP) is an example of next generation sequencing (NGS) tool that allows for rapid, cost-effective, high resolution genotyping of multiple specimens in parallel. The hundreds of reads obtained for each specimen can be used to identify minor variants or be integrated to approximate Sanger sequencing (SS) genotyping [4], [16]. We used the high resolution TPP to genotype DBS, circulating and proviral HIV from a cohort of patients with varied ART exposure, duration of infection and viral load (VL) in order to determine whether DBS genotypes remain equivalent to plasma genotypes with NGS methods.

## Materials and Methods

### Ethics Statement

This research involves only anonymized clinical specimens and the relevant research protocol had been approved by the Ottawa Hospital Research Ethics Board (OHREB). All participants provided their written informed consent to participate in the study.

### Subjects and specimen

After informed consent, 17 HIV-1 positive subjects provided an EDTA anti-coagulated blood specimen. A FACSCalibur flow cytometer (BD Biosciences, USA) was used for CD4^+^ cell enumeration. DBS were prepared by pipetting 75 µl/spot of whole blood onto Whatman 903® filter paper (Whatman Inc, Florham Park, USA). Each card was air-dried overnight at room temperature, individually packaged in a zip-lock bag and stored at −20°C with desiccant. After centrifugation, plasma was transferred to a fresh tube and the pellet re-suspended and used for peripheral blood mononuclear cells (PBMC) isolation using Ficoll-Hypaque density gradient centrifugation. Nucleic acid (NA) was extracted from two DBS spots and from 200 µl of plasma using the Nuclisens EasyMag system (Biomerieux, Canada) following manufacturer's instructions. Cellular DNA was extracted from the isolated PBMCs using QIAamp DNA Mini Kit (Qiagen, Mississauga, Canada). Plasma VL was measured using the Versant HIV RNA 3.0 Assay (bDNA, Siemens Healthcare Diagnostics, Mississauga, Canada).

### HIV Genotyping by TPP

TPP was performed on PCR amplified, multiplex identifier (MID) labeled amplicons, covering the protease (PR) and reverse transcriptase (RT) (partial) genes from NA extracts derived from DBS, plasma, or PBMC following published methods [4].

### Sequence analysis

TPP reads were screened for quality using the GS FLX defaults and decoded using Roche Amplicon Variant Analyzer software. Reads passing initial QC were re-screened using custom Perl scripts to further improve the accuracy of the downstream analysis [4], [16], [17] (Figure S1). In brief, the reads were the first filtered using the following criteria: 1) a read length of ≥100 bps; 2) an average quality score of ≥25; 3) no ambiguous bases present in the read. All valid reads were then mapped to the HXB2 reference (GenBank Accession: K03455) by BLAST. Only reads that had ≥65% overlap and ≥75% identity with HXB-2 reference were employed to generate multiple alignments on assumption that there are not true insertions. The net PCR and pyrosequencing error rates were estimated by parallel pyrosequencing three pedigreed plasmid controls.

Sequence contigs for each specimen were built using all the valid reads which were aligned against HXB-2. Two consensus sequences were generated for each specimen with mixed base identification thresholds (MBIT) of 5% and 20% respectively. The MBIT defines the threshold for calling a minor variant based upon the frequency of the mutation at a specific locus within the aligned individual pyrosequencing reads. A 5% MBIT was chosen as the reference consensus sequence in order to maximize our ability to detect discordance among the different specimen formats. Discordant base positions from any of the three specimen formats from the same subject were flagged. These flagged positions were then used to evaluate the overall inter-format sequence concordance rates (SCR) among specimens by using the 20% MBIT to simulate the readout from conventional genotyping. The derived SCRs were analyzed using SPSS 12.0, stratified according VL, CD4 counts, ART exposure or duration of HIV infection. Both 5% and 20% MBIT consensus sequences were also examined for transmitted HIV drug resistance (TDR) using the CPR tool 4.1 (URL: http://cpr.stanford.edu/cpr) and results were compared across formats.

## Results

Most subjects were born in North America and infected with subtype B HIV-1 virus. The single African subject was infected with a subtype C virus. Twelve subjects were ART-naïve, 3 subjects were receiving ART, while 2 had received ART therapy in the past. The duration of HIV infection, was between 2 months and 20 years. Subjects had a detectable VL with the range 504 ∼50,192 copies/ml. Only 3 of 17 subjects had CD4 counts ≤200 cells/µl. (Table 1).

**Table 1:** Demographic and clinical characteristics data of subjects in the study cohort.

Subjects	Viral Load (Copies/ml)	CD4 Count (per µl)	Sex^a^	Age Group	Birth Place^b^	HIV-1 Subtype	Duration of Infection (yr)	ART status^c^
013	6,080	282	M	40–60	N. A	B	2.0	ART naive
020	3,406	587	M	18–25	N. A	B	5.5	ART intermittence
022	9,356	250	M	40–60	S. A	B	8.0	ART naive
024	38,658	316	M	>60	N. A	B	6.5	ART naive
029	43,750	307	M	25–40	Africa	C	4.5	ART naive
036	5,748	535	F	18–25	N. A	B	1.0	ART naive
038	46,896	450	M	25–40	N.A	B	5.0	ART naive
039	2,378	108	M	40–60	N.A	B	20.0	Kaletra, Truvada
042	1,448	484	F	40–60	N.A	B	18.0	ART naive
049	7,352	185	M	25–40	S.A	B	0.2	ART naive
058	2,278	499	M	25–40	N.A	B	5.0	Kivexa, ATV, RTV
064	35,774	444	M	40–60	N.A	B	0.1	ART naive
065	14,240	324	M	40–60	N.A	B	4.5	ART naive
067	11,076	256	M	40–60	N.A	B	14.0	ART naive
080	4,462	208	M	25–40	S.A	B	0.5	ART naive
083	504	49	M	40–60	N.A	B	14.0	DRV, RTV, ETR, Truvada
086	50,192	115	M	40–60	N.A	B	14.0	ART intermittence

Abbreviations:

a : M: Male; F: Female.

b : N.A: North America; S.A: South America;

c : ATV: Reyataz; RTV: Norvir; DRV: Darunavir; ETR: Etravirine; Truvada.

Plasma, PBMCs and DBS for all subjects were successfully sequenced by TPP. A total of 49,222 TPP reads were used in downstream analysis. The average oversampling rate was 290 for each nucleotide position for the 17 sets of specimens. The net TPP error rate was 0.35% as measured using pedigreed plasmid controls.

Nucleotide variations identified at the 5% MBIT level were re-evaluated through pairwise comparison among plasma, PBMCs and DBS, using consensus sequences generated with an MBIT of 20% [4], [18]. The average SCR for each subject were 82.9±11.9% for DBS vs. plasma, 78.9±10.9% for DBS vs. PBMC and 75.31±14.7% for plasma vs. PBMC.

**Figure 1:**
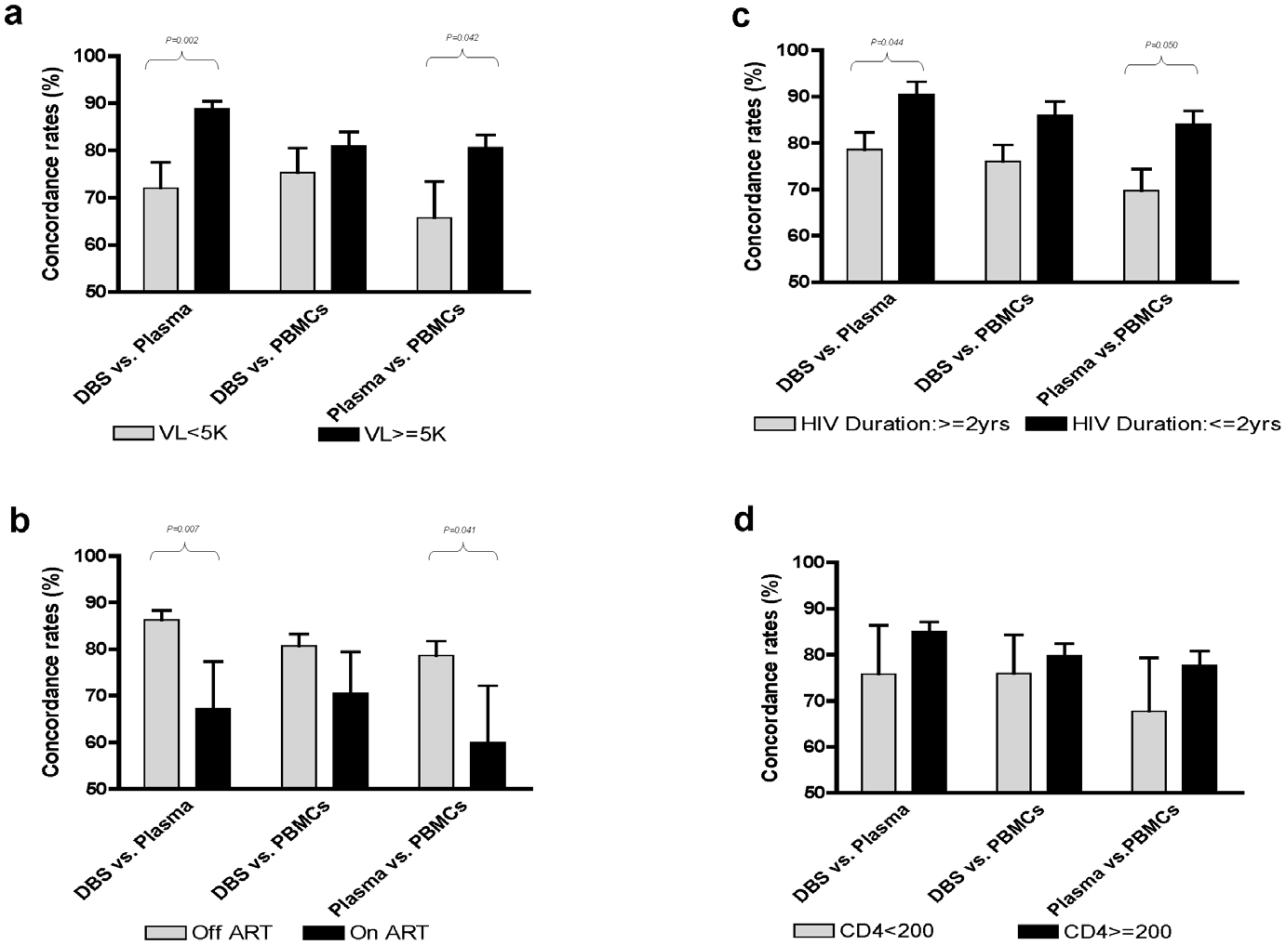
Inter-format concordance rates in HIV-1 genotypes acquired from plasma, DBS and infected PBMCs. The inter-format sequence concordance rates (SCR) (mean ! SD) among patients with varied clinical status were plotted and p values of pairs with statistically significant differences were depicted as well. Significantly higher SCRs between genotypes from plasma and DBS or PBMCs were observed when VL is higher than 5,000 copies/ml (a) or when the patients were off antiviral therapy, either treatment-naïve or during therapy intermittence (b) or when the duration of HIV infection is shorter than 2 years (c).However, CD4 count should little impact on the SCR among genotypes acquired form the three specimen formats (d). None of the factors showed significant impact on the SCR level between DBS and PBMCs.

SCR were re-analyzed after subjects were stratified according to VL, ART status, CD4 counts, and duration of HIV infection. When compared to subjects with a VL of >5,000 copies/ml, subjects with a lower VL had a greater sequence discordance between plasma and either DBS or PBMC. The mean SCR for DBS vs. plasma was 72.0% when the VL was <5,000 copies/ml, and 88.8% when the VL was ≥5,000 copies/ml (p = 0.002). The plasma vs. PBMC SCR was 65.7% vs 80.6% (p = 0.042) in the same low/high viral load stratification. No significant difference in the SCR was found when comparing sequences from DBS and those from PBMC at different VL (75.3% vs 80.9%, p = 0.325) (Figure 1a).

Current ART use also had a significant impact on the SCR. When stratified according to ART status, the mean SCRs in the off-ART group (ART-naïve or prior ART) were 86.3%, 80.7% and 78.6% for the pairs of DBS vs. plasma, DBS vs. PBMC and plasma vs. PBMC respectively. In contrast, the corresponding SCRs in the on-ART groups were 67.2%, 70.5% and 59.9% respectively. The inter-group differences were statistically significant for plasma vs.DBS and plasma vs. PBMCs pairs (p = 0.007- and 0.041 respectively). Although not statistically significant, the DBS vs. PBMC comparison showed a trend to greater differences in SCR when the patients were off-ART (p = 0.146) (Figure 1b). Although ART exposure appeared influences SCR, multivariate analysis demonstrated that VL level was the only independent correlate of SCR when comparing DBS with plasma.

The duration of HIV infection was correlated with the SCRs found when the specimens were compared (Figure 1C). For example, a significantly higher SCR between DBS- and plasma-based HIV genotypes were observed in patients with an infection of ≤2 years as compared to those infected by HIV for longer periods (90.5% vs 82.8%, p = 0.044) . This finding was also seen when plasma and PBMC genotypes were compared. (SCR 84.0% vs 74.8%, p = 0.05). The same SCR trend was observed for the DBSvs. PBMC comparison although the difference was not significant (p = 0.08).

Absolute CD4 counts had little impact on the SCRs. When comparing patients with CD4 counts <200 cells/µl and those with ≥200 cells/µl, the SCRs for the DBS vs. plasma, DBS vs. PBMC and plasma vs. PBMC were 75.8% vs 85.1%, 76.0% vs 79.9% and 67.7% vs 77.6% respectively. Although all the SCRs tended to be higher while the CD4 counts increased, these differences were not statistically different regardless of the whether a threshold of 200 cells/µl (Figure 1c) or 350 cells/µl was used [19] (data not shown).

With VL, ART status and the duration of HIV infection all identified as correlates with the inter-format SCRs, we conducted stepwise regression analysis aiming to identify the independent determinant(s) that best predicted the genotype concordance. The results showed that the VL was the sole independent factor that predicts the SCR between DBS and plasma regardless of which MBIT cutoff (5% or 20%) was applied (F = 13.698 and p = 0.002 for MBIT20, and F = 12.308 and p = 0.003 for MBIT5) . Higher VLs were always associated with greater concordance between DBS and plasma genotypes. In contrast, the ART status was revealed to be the only independent predictor for plasma vs. PBMC concordance (F = 4.66 and p = 0.047 for MBIT20). The absence of ART (ART-naïve or prior ART) predicted higher HIV genotype concordance between plasma and PBMCs regardless of their VL levels or duration of HIV infections.

When we evaluated the inter-format concordance at drug resistance mutation (DRM) sites, we noted that identical mutations were detected in all specimen formats in one patients (Subject 013, ART-naïve, VL: 6,080 copies/ml, CD4 count: 282 cells/µl); in plasma and DBS only for a second patient (Subject 039, on ART with Kaletra and Truvada, VL: 2,378 copies/ml, CD4 count: 108 cells/µl); and only in PBMC in the third (Subject 083, on ART with Darunavir + Ritonavir + Etravirine, VL: 504 copies/ml, CD4 count: 49 cells/µl). Although there were only a small number of subjects with DRMs, DBS results were concordant with plasma results.

## Discussion

Implementation of DBS as a collection and storage method for HIV genotyping is predicated upon the results from DBS and plasma being concordant [2], [4], [18], [20]. Most data demonstrating genotype concordance are based upon studies of ART naïve subjects which would be expected to have a consistent viral genotype in all compartments [21]. Furthermore, the comparisons were all conducted based on the examination of the overall sequence similarity using bulk sequencing. However, the homogeneity of HIV genotypes among cell-free and cell-associated subpopulations undergoes dynamic changes while the infection progresses and especially under ART [8]. The proximal immune and/or ART selection pressures may exert distinct evolutional influence on the circulating viruses and the archived proviral population resulting in discordant HIV genotypes. DBS genotypes, made from whole blood, are genetic mosaics of circulating virus from plasma, and the cell associated archived provirus. During the course of infection, factors \may influence the quantity of the viruses present from each compartment that may affect the measured DBS genotype. This state is further complicated by the relative instability of the viral RNA on DBS, when present at lower copy numbers, which further alter the ratio of circulating to integrated genotypes [22]. Using deep pyrosequencing, we were able to examine, in high resolution, the accuracy of DBS genotypes with reference to plasma and PBMC among patients with varied VLs, CD4 counts, ART exposure and durations of HIV infection.

Our data clearly demonstrate that the VL, ART status and duration of HIV infection are factors that correlate with the concordance of HIV-1 genotypes between plasma and DBS or PBMCs. Subjects with VL ≥5,000 copies/ml or who were ART naive or with the duration of HIV infection of ≤2 years were found to have significantly higher concordance among the DBS/plasma and PBMC/plasma pairs. Multivariate analysis revealed VL as the independent determinant influencing SCR between DBS and plasma with ART status and duration of HIV infection as surrogates for this correlation. Consistent with the differences between DBS and plasma genotypes, we found differences between DBS and PBMC genotypes, however, these latter differences did not reach statistical significance. If DBS were solely composed of cellular material then the sequencing results would be expected to be identical to those from PBMC's. Given that DBS genotypes are the result of the merging of proviral and circulating viral sequences, then finding the DBS/PBMC genotypes not statistically different seems odd. However, one possible explanation is that variability in circulating viral sequences measured on DBS was sufficiently attenuated by the averaging effect of the co-sequenced provirus that it was difficult to demonstrate any statistical difference from PBMC sequences.

The significant role of VL as a predictor of genotype concordance between DBS and plasma specimens is consistent with three lines of evidence. Monleaus' finding that a VL is an important predictor or genotyping success demonstrates that circulating RNA plays an important role in drug resistance testing from DBS [23]. Steegen et al suggested that, at lower viral loads, there may a difference in genotypes obtained from circulating virus as compared with genotypes from cellular DNA [24]. Third, it has been well described that proviral genotypes can vary significantly from those detected in the plasma [13], [15], [25]. With the addition of evidence from our study, we suggest that DBS most accurately approximate plasma genotypes at higher viral loads. One explanation is that concordance is dependent on the presence of more labile circulating viral RNA that is subject to more rapid degradation than DNA on DBS. With a viral half-life on the order of 1 day [26], higher viral loads are consistent with an equilibrium established by the massive, ongoing exchange of virus between cellular compartment and the circulation. Thus it is not surprising to find that where DBS contain large initial amounts of circulating viral RNA the concordance with plasma genotypes is higher.

In contrast to previous studies that demonstrated DBS/plasma genotype equivalency among treated patients [27], we show that, ART results in DBS genotypes becoming less concordant with those from plasma. The effect of ART exposure on concordance was subject to the effect of viral load; with those subjects having high viral loads and ART exposure remaining more concordant. Our findings that ART exposure independently predicts discordance between plasma and PBMC genotypes hints at the origin of the differences in DBS/plasma genotypes in the ART exposed. Given that with ART there may be sequence differences in archived provirus when compared to the circulating virus, at lower viral loads, the discordance between DBS and plasma would be expected to be more pronounced. Our data also support the converse, in that while ART is in use where VL remains high, the contribution of circulating virus to the DBS composite sequence resolves the plasma/PBMC discordance. The specific VL at which subjects failing ART have DBS genotypes becoming discordant with their plasma genotypes remains to be determined.

The trend of sequence concordance in the absence of ART exposure remained valid when DRM were examined. The only subject in whom plasma, DBS and PBMC TDR results were concordant was ART naïve; while the two individuals with discordant TDR results were on ART. Previous studies from our group and the others demonstrated that DBS generated HIV DR typing results comparable to those from plasma in ART naïve patients. However, among treatment experienced subjects, despite demonstration of overall concordance in DR, the only clear discordance at a major mutation site arose from a specimen with a viral load of <5,000 copies/ml which is consistent with our findings [27]. Furthermore, our results are consistent with other groups who found that 1/3 of the DBS specimens were discordant with the plasma specimens when minor variants were examined [15], [28]. In the context of increasing interest in minority variants, we would predict that the differences between DBS and plasma genotypes will become more apparent.

Limitations of this study include the limited number of subjects harbouring HIV DRM. While correlations in DBS vs. plasma sequence discordance and VL were clear, we did not have the power to make definitive statement on similar trends in DR. Second, although we found that viral load was an independent predictor of concordance, a larger sample set may help to determine the exact role of ART in its influence on VL as well as the genotype itself. Thirdly, we used a 5% MBIT to identify variable regions and create the denominator for the analysis, which increased the amount of observed variance. This approach was chosen as it filters for those genomic regions that are presumably being actively selected, either by the host or drug pressure which are more likely to be detected by NGS methods. While a larger sample size may prove more robust in establishing a correlation, our study was limited by the availability of specimens, and technical limitation involved in obtaining sufficient multiple reads over all specimen formats for each subject. Despite this, a consistent trend of lower concordance among DBS, plasma and PBMCs was observed in all ART exposed patients regardless of their VL levels, suggesting that our data interpretation remains valid despite of smaller number of ART experienced subjects.

In conclusion, our results suggest that DBS genotypes most closely resemble the plasma genotype when the plasma VL is ≥5,000 copies/ml and/or the patient is ART naïve and/or the HIV infection is still at earlier stage. Among treatment experienced patients, the sequence discordance suggests that DBS genotypes may not consistently represent those from plasma. It is possible that these findings may limit the application of DBS specimens for monitoring of patients on ART, especially in the context of more sensitive next generation sequencing genotyping methods.

## Supporting Information

Figure S1 **Workflow of extended TPP read quality screening and data processing.**
(DOC)
