# Supplementary material for: Pyrosequencing Dried Blood Spots Reveals Differences in HIV Drug Resistance between Treatment Naïve and Experienced Patients
Source: PLoS One. 2013 Feb 7;8(2):e56170. doi: 10.1371/journal.pone.0056170 (PMC3567018; doi:10.1371/journal.pone.0056170)
Supplement: Figure S1 — Workflow of extended TPP read quality screening and data processing. (DOC) [file pone.0056170.s001.doc]

Filter (>100 bps; Average Score >25)

Raw Reads Good Reads

Bad Reads

Mapping to

(by BLAST)

HXB2 Reference

(PR/RT)

Record of coordinates of reads

(Cut-off: >65% overlap; >75% identity)

Generation of multiple alignments

(Coordinates to reference)

Consequence sequence generation

(MBIT 5 and MBIT20)

**Figure S1 Workflow of extended TPP read quality screening and data processing**
